# Junctional ER Organization Affects Mechanotransduction at Cadherin-Mediated Adhesions

**DOI:** 10.3389/fcell.2021.669086

**Published:** 2021-06-17

**Authors:** Michelle Joy-Immediato, Manuel J. Ramirez, Mauricio Cerda, Yusuke Toyama, Andrea Ravasio, Pakorn Kanchanawong, Cristina Bertocchi

**Affiliations:** ^1^Laboratory for Molecular Mechanics of Cell Adhesion, Department of Physiology, Faculty of Biological Sciences, Pontificia Universidad Católica de Chile, Santiago, Chile; ^2^Institute of Biomedical Sciences, Faculty of Medicine, Universidad de Chile, Santiago, Chile; ^3^Center for Medical Informatics and Telemedicine, Faculty of Medicine, Universidad de Chile, Santiago, Chile; ^4^Mechanobiology Institute, National University of Singapore, Singapore, Singapore; ^5^Department of Biological Sciences, Faculty of Science, National University of Singapore, Singapore, Singapore; ^6^Institute for Biological and Medical Engineering, Schools of Engineering, Medicine and Biological Sciences, Pontificia Universidad Católica de Chile, Santiago, Chile; ^7^Department of Biomedical Engineering, Faculty of Engineering, National University of Singapore, Singapore, Singapore

**Keywords:** cadherin-mediated adhesion, mechanotransduction, endoplasmic reticulum, microtubules, vinculin

## Abstract

Cadherin-mediated adhesions (also known as adherens junctions) are adhesive complexes that connect neighboring cells in a tissue. While the role of the actin cytoskeleton in withstanding tension at these sites of contact is well documented, little is known about the involvement of microtubules and the associated endoplasmic reticulum (ER) network in cadherin mechanotransduction. Therefore, we investigated how the organization of ER extensions in close proximity of cadherin-mediated adhesions can affect such complexes, and vice versa. Here, we show that the extension of the ER to cadherin-mediated adhesions is tension dependent and appears to be cadherin-type specific. Furthermore, the different structural organization of the ER/microtubule network seems to affect the localization of ER-bound PTP1B at cadherin-mediated adhesions. This phosphatase is involved in the modulation of vinculin, a molecular clutch which enables differential engagement of the cadherin-catenin layer with the actomyosin cytoskeleton in response to tension. This suggests a link between structural organization of the ER/microtubule network around cadherin-specific adhesions, to control the mechanotransduction of adherens junctions by modulation of vinculin conformational state.

## Introduction

Adherens junctions (AJ) are adhesion complexes between neighboring cells recently identified as mechanosensors that transduce changes in tissue tension into biochemical signals to contribute to the physiological processes occurring during morphogenesis, cell growth, and differentiation (Green et al., 2010; Niessen et al., 2011; Leckband and de Rooij, 2014). AJs connect the actin cytoskeleton of adjacent cells through transmembrane cadherin receptors and a network of adaptor proteins, collectively known as the cadhesome (Bertocchi et al., 2012; Zaidel-Bar, 2013). Similarly to integrin-based adhesions (also known as focal adhesions, formed between cell and extracellular matrix), this network is organized into a stratified nanoscale architecture with components layered into three main compartments (Han and De Rooij, 2016; Bertocchi et al., 2017; Xia and Kanchanawong, 2017). Within this architecture, the cadherin-catenin layer at the membrane and the actin regulator layer are separated by what we named the “interface zone,” that contains vinculin, a stretchable molecule which has long been reported to be implicated in cells’ ability to sense mechanical forces (le Duc et al., 2010; Peng et al., 2011; Xia and Kanchanawong, 2017; Bertocchi et al., 2019). Vinculin, thanks to its anchorage to α-catenin and to its strategic position in the interface force-transduction zone, undergoes dramatic shape-shifting transformation from a compact conformation to a highly elongated form, serving the role of a molecular clutch capable of differentially engaging cadherin with the actin cytoskeleton in response to tension and/or tyrosine phosphorylation (Han and De Rooij, 2016; Bertocchi et al., 2017).

Despite the growing information gathered around the role of the actomyosin cytoskeleton in mechanotransduction at cadherin-mediated adhesion, the potential link between the microtubule and the associated endoplasmic reticulum (ER) network with cadherin-mediated adhesions has been less extensively investigated (Terasaki et al., 1986; Terasaki and Reese, 1994). The ER (Ozawa et al., 1990; Hinck et al., 1994; Chen et al., 1999) and the microtubules (Mary et al., 2002; Dogterom and Koenderink, 2019) are known to play a role in promoting junction formation by mediating targeted delivery of adhesion components to the plasma membrane and by facilitating local recruitment and activation of myosin II, to finally induce accumulation and cluster of E-cadherin for adhesion assembly (Stehbens et al., 2006). Nevertheless, the cooperativity between cadherins and the ER in cadherin-mediated adhesion mechanotransduction is still largely neglected (Stehbens et al., 2009). Interestingly, PTP1B, a non-receptor protein tyrosine phosphatase, previously reported to be involved in mechanotransduction at cadherin-mediated adhesions, is anchored to the cytoplasmic side of the ER (Frangioni et al., 1992). While it dephosphorylates and inactivates receptor tyrosine kinases, as they transit by the ER (Haj et al., 2002), it appears to also be able to access some plasma membrane-bound substrates specifically at points of cell–cell contact (Anderie et al., 2007). In line with this, it was demonstrated that the ER tubular network can come into close proximity to the plasma membrane at specialized regions of cell–cell contact, thus potentially enabling PTP1B to engage with substrate(s) at these sites (Anderie et al., 2007), and having a role in regulation of cell adhesion. For instance, PTP1B has been described as a positive regulator of c-Src kinase activity, being the primary protein tyrosine phosphatase capable of dephosphorylating the inhibitory Y529 site in Src, thereby activating this kinase for AJ and focal adhesion assembly and disassembly (Bjorge et al., 2000; Yip et al., 2010). In our previous studies, we have shown the involvement of PTP1B in the dephosphorylation of vinculin. We have observed that PTP1B-mediated dephosphorylation maintains vinculin in a compacted, partially unfurled conformation, in lower tensional E-cadherin-based adhesions [in Madin-Darby canine kidney (MDCK) cells], whereas, in the higher tensile N-cadherin-based adhesions (in C2C12 myoblasts), vinculin may be fully activated due to both higher contractility and lower dephosphorylation induced by PTP1B (Bertocchi et al., 2017). Still, it remains unknown whether this difference reflects E-/N-cadherin specificity, and whether it is due to a physical inaccessibility of PTP1B to the N-cadherin-based junctions, possibly due to a structural difference of the ER/microtubules in different cell types and their associated different tensional states.

In the present paper, we show that the access of the ER to the AJ appears to be tension dependent. In epithelial cells, the ER extends to the low tensional E-cadherin-mediated adhesions. In C2C12 cells, the ER is not able to penetrate the higher tensile N-cadherin-mediated adhesions, unless tension is released. This could suggest a possible role for ER organization in the positioning of PTP1B at sites of adhesion, and it would explain how the difference observed in conformational state of vinculin in the two different cell types is intertwined with structural differences in the ER/microtubule organization around E- and N-cadherin-mediated adhesions, to ultimately control mechanotransduction of AJs.

## Materials and Methods

### Cadherin Planar Biomimetic Substrate Preparation

Two different types of support have been used for the preparation of the cadherin planar biomimetic substrate (a model of this substrate can be found in Supplementary Figure 1): coverglasses (#1.5, 18 mm diameter) and silicon oxide wafers, p-type (100)-orientation with 500 nm thermal oxide (Bondatek; Addison Engineering). The glass coverslips were initially UV sterilized for 15 min and washed with DPBS under a sterile tissue culture cabinet, while the silicon wafers were cleaned following the procedure described previously (Bertocchi et al., 2017). Briefly, wafers cut into squared chips of 1.2 cm^2^ with a diamond-tip pen were sequentially washed in distilled water (dH_2_O), then sonicated in 100% acetone for 20 min, washed in dH_2_O, sonicated in 1 M potassium hydroxide for 20 min, and washed in dH_2_O to remove any organic residue. Either support (coverglasses or silicon wafers) underwent silanization with 3-glycidoxy-propyl-dimethoxymethylsilane (Sigma) (0.045% in ethanol 100%) to allow for chemical protein conjugation. After 1 h at RT on a horizontal shaker, the coverglasses and the wafers were cured for 1 h at 110°C in a static oven. Silanized substrates were then washed with 70% ethanol and dH_2_O, before being air-dried and stored until usage. Silanized substrates were incubated, the day before the experiment, with anti-Fc fragment antibody (in 0.1 M, pH 8 borate buffer) directed versus E-cadherin-Fc or N-cadherin-Fc fusion chimera (Jackson ImmunoResearch, West Grove; #115-005-008 and #115-005-071, respectively), at a final density of 1 g/cm^2^ in a self-assembled humidity-controlled chamber at 4°C o.n. After incubation, the silanized substrates, coated with anti-Fc-antibodies were washed in PDBS and blocked with 100 mM NaHCO_3_ (pH 8.3) and aminoethoxy-ethanol (Sigma) for 1 h at RT. Following neutralization, the substrates were treated with specific E- or N-cadherin-Fc chimera proteins (R&D system; #648-EC-100 and #6626-NC-050, respectively) at a final density of 1 g/cm^2^ for 2 h at RT washed with PBS with Ca^2+^ and Mg^2+^ (to avoid any effect on cadherin structure), and passivated by using 0.2% pluronic acid (Sigma) at RT for 20 min.

### Cell Culture

MDCK and Eph4 (mouse mammary gland epithelial) cells were both maintained in Dulbecco’s modified Eagle’s medium (DMEM) supplemented with 10% fetal bovine serum (FBS) and 100 units/ml of penicillin/streptomycin (Life Technologies). C2C12 cells were cultured with DMEM supplemented with 20% FBS and 100 units/ml of pen/strep. Cell transfection was performed by electroporation using the Neon transfection system (Life Technologies) according to the manufacturer’s protocol; 1–10 μg endotoxin-free plasmid DNA was used for 1 × 10^6^ cells. No cell lines used in this study were found in the database of commonly misidentified cell lines maintained by ICLAC and NCBI Biosample. The cell lines were not authenticated. All cell lines used for this study were maintained mycoplasma free as regularly tested by PCR methods.

After transfection, cells to be plated on cadherin biomimetic substrates were trypsinized and seeded in serum-free medium onto the proper biomimetic cadherin substrate (coverglasses or silicon wafers) and incubated in a humidified atmosphere at 37°C and 5% CO_2_ in for 4–6 h. For laser ablation experiments, cells were seeded on coverglasses precoated with bovine fibronectin (F1141, Sigma) at a concentration of 10 μg/ml.

### Fluorescent Protein Fusion Constructs

Plasmids for vinculin wt (N-terminus-eGFP and C-terminus-tdEOS), calnexin-mEmerald, and ZO1-mEmerald were obtained from the laboratory of MW. Davidson (The Florida State University), and they are commercially available on Addgene repository. Y1065E, Y1065F, Y822E, and Y822F mutants of vinculin were generated by MBI Protein Expression Facility (Singapore), as described previously (Bertocchi et al., 2017). Src Y529F is commercially available from Addgene (#17686). Localization of the indicated fluorescent proteins (FPs) to AJ was assessed in cells plated on fibronectin and cultured to confluence, as in Bertocchi et al. (2017).

### Immunostaining

Cells were fixed with 4% PFA (paraformaldehyde) for 15 min at 37°C in a humidified chamber, followed by blocking with 10% FBS and permeabilization with 0.2% Triton X-100 for 2 min. Samples were incubated 1 h at RT with the following primary antibodies, at the indicated dilutions: rat anti-E-cadherin (Abcam #ab11512) 1:1,600; rabbit anti-β-catenin (Abcam #ab16051) 1:400; rabbit anti-tubulin (Abcam #ab18251) 1:500; rat anti-tubulin (GeneTex #GTX76511) 1:500; mouse anti-PTP1B (Abcam #ab124375) 1:400. After washing and blocking, samples were incubated in a humidified atmosphere with secondary antibodies at a dilution of 1:400: donkey anti-mouse and goat anti-rabbit, conjugated with Alexa Fluor 488 and Alexa Fluor 568 (Life Technologies). Phalloidin Alexa Fluor 647 (Thermo Fisher Scientific #A22287) was used at a dilution of 1:200. Cells on biomimetic substrates were fixed for imaging ∼4–6 h after replating, using 4% paraformaldehyde and stored in PHEM buffer (PIPES, 60 mM; HEPES, 25 mM; MgCl_2_, 2 mM; without EGTA, pH 7.0).

### Scanning Angle Interference Microscopy

Nanoscale precision Z-position measurement by surface-generated structured illumination was performed as detailed in Bertocchi et al. (2017, 2020). The details of the optical theoretical foundation of scanning angle interference microscopy (SAIM) were described previously (Paszek et al., 2012; Bertocchi et al., 2020). Image acquisition was performed on a Nikon Eclipse Ti inverted microscope (Nikon Instruments), with a motorized TIRF illuminator, using a × 60 ApoTIRF objective with NA 1.49, a sCMOS camera (Orca Flash 4.0, Hamamatsu). The thickness of the oxide (used as a spacer to create interference) of each silicon wafer was measured by ellipsometry (UV-VIS-VASE) allowing for nanometer precision.

The cells seeded on the cad-Fc substrate on the silicon wafer were placed face down in a PBS-filled 27-mm glass-bottom dish (Iwaki). Fluorescence images were acquired every 4° between 0 (normal) and 52° (critical angle), as described previously (Paszek et al., 2012; Bertocchi et al., 2020). For vinculin-c-tdEOS, photoconversion was achieved through excitation with 405 nm LED. Analysis of Z-position mapping was obtained by an IDL-based software; initial image thresholding was followed by generation of binary masks for cadherin-mediated adhesions, indicated as regions of interest (ROIs). To determine the topographic height (*Z*), Levenberg Marquardt non-linear least-square fit was used with multiple initial guesses for exhaustive search, for each pixel in the binarized ROI (Paszek et al., 2012; Bertocchi et al., 2017, 2020). The representative Z-position for each ROI was indicative of its median Z-position value and indicated as *Z*_center_. The *Z*_center_ values of all ROIs of one specific protein were pulled together to obtain a frequency distribution, the median of which represented the protein position, indicated as *Z*_median_.

### Laser Ablation of Cell–Cell Junction in Epithelial Monolayers

Following the same protocol indicated in Bertocchi et al. (2017), tension at cell–cell junction was assessed in MDCK epithelial monolayers by laser ablation with a UV laser using 15 nW laser power focused on the back aperture of the objective lens on a Nikon A1R MP laser scanning confocal microscope with 350 ms exposure time. Images were acquired every 2 s, starting three frames before ablation for 33 frames in total. Analysis of recoil speed consisted in the tracking of the two edges of the cut in the sequential frames, and it can be easily performed by MTrackJ plug-in (Meijering et al., 2012) in ImageJ. The recoil speed (expressed as ms^–1^) is defined as the rate of movement (position change) of the two edges in the time.

### Drug Treatment

For pharmacological inhibition, cells were treated using the following concentration of drugs prior to sample fixation or laser ablation experiment: sodium orthovanadate (S6508, Sigma) 100 μM, RK682 (RK2033, Sigma) 10 μg/ml; ALLN (ab141445, Abcam) 10 and 50 μM, ALLM (ab141446, Abcam) 10 and 50 μM, nocodazole (M1404, Sigma) 10 μM, and Y-27632 (Y0503, Sigma) 10 μM.

While sodium orthovanadate is a generic alkaline and tyrosine phosphatase inhibitor, RK682 is a specific and non-competitive inhibitor of protein tyrosine phosphatase with confirmed low micromolar inhibitory activity against protein tyrosine phosphatases CD45 and PTP1B, and dual-specificity phosphatases VHR, CDC-25A-B-C (Hamaguchi et al., 1995); nevertheless, CD45 and VHR genes are not expressed in MDCK and EpH4 cells, as indicated by RNAseq data (Supplementary Table 1), while inhibition of CD25 requires longer incubation period (20 h) with respect to what we have used in the current paper (1 h).

ALLN and ALLM are inhibitors for calpains, with high binding efficiency for calpain-1 and calpain-2, respectively. ALLN has been reported to inhibit the calpain/PTP1B axis in endothelial cells (Zhang et al., 2017).

### Statistical Analysis

Plots and statistics for Z-position measurements, and laser ablation data (one-way ANOVA, followed by pairwise Tukey test) were performed using Prism 8.0.1 (GraphPad) and OriginPro software. Mean, standard deviation, SEM, and *n* of laser ablation experiments are shown in Figure 5. Differences were considered significant when *P* < 0.05. For protein Z-position, data are presented as medians (nm) indicated below each box plot in each graph in Figures 6A,C,E; numbers of adhesions (number of ROIs) and the numbers of cells (*n*_cells_) are shown above each box plot. Immunofluorescence images are representative of at least three independent samples, and they are presented with the quantification of the entire data set. Numbers indicative of experiment replication are indicated in the corresponding graphs and in their legends.

## Results

### Microtubules and the Endoplasmic Reticulum Are Directed Toward E-Cadherin-Mediated Cell–Cell Contacts

**FIGURE 1:**
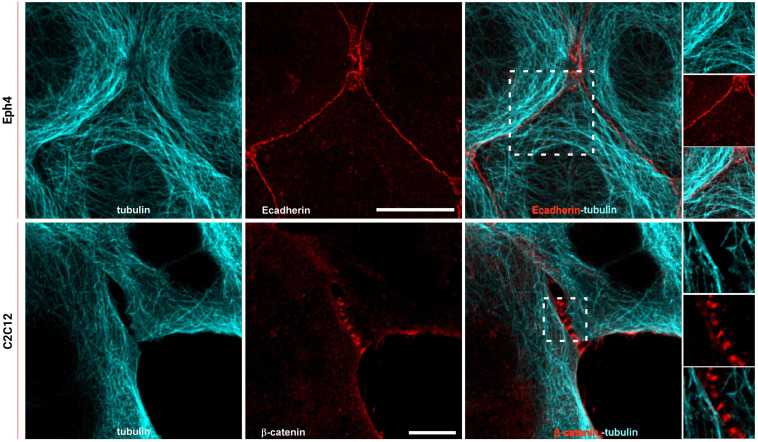
Microtubules reach cadherin-mediated adhesions in epithelial cells but are maintained at a distance in C2C12 cells. Top: Eph4 cell monolayers labeled for tubulin (cyan) and E-cadherin (red); bottom: C2C12 cells labeled for tubulin (cyan) and β-catenin (red). Images represent microtubule staining alone, E-cadherin or β-catenin, and the merged image. In the zoom image (white box in the merge image on top panel), the overlapping of the microtubule network (cyan) with E-cadherin-mediated adhesion (red) is visible. In the zoom image (white box in the merge image on the bottom panel), it is visible that the extension of the microtubules (cyan) in C2C12 do not reach the adhesions (β-catenin in red). Images on the top panel were acquired by spinning disk confocal microscope with a 100x objective, 1.49NA Plan-Apo (Nikon). Images on the bottom panel were acquired by LSM 880 confocal microscope with Airyscan detection with a 60x objective, 1.4NA Plan-Apo (Zeiss). Scale bars, 10 μm.

**FIGURE 2:**
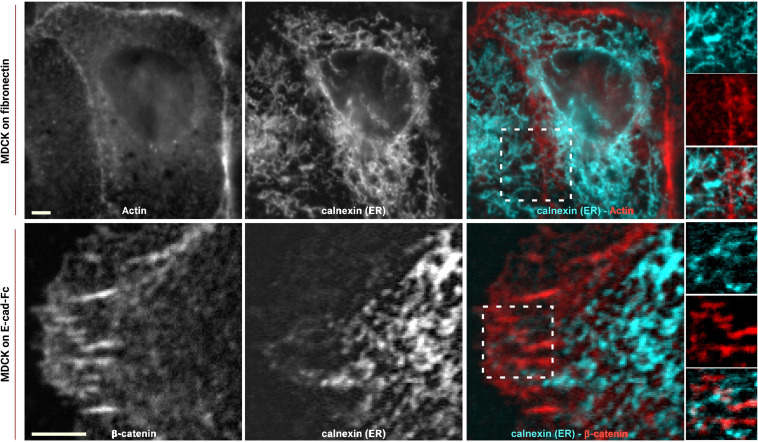
ER reaches E-cadherin-mediated adhesions in epithelial cells. MDCK cells transfected with calnexin-mEmerald seeded on fibronectin (top) and on E-cadherin biomimetic substrate (bottom) and stained with phalloidin or β-catenin (red), respectively. Images represent ER alone (left), phalloidin or β-catenin (center), and the merged image (right). In the enhanced contrast images (corresponding to the white boxes in the merged images), the overlapping of the ER with the cadherin-mediated adhesion is clearly visible, in both systems, on fibronectin (top panel) and on E-cadherin biomimetic substrate (bottom panel). Images were acquired by spinning disk confocal microscope with a 60x objective, 1.49NA Plan-Apo (Nikon). Scale bar, 5 μm.

To study how the organization of ER extensions in close proximity of cadherin-mediated adhesions can affect such complexes, we firstly assessed the capability of the microtubule extensions (on which the ER runs; Fuentes and Arregui, 2009) to reach cell–cell contacts. Using epithelial EpH4 cells (Figure 1, top panel) and myoblast C2C12 cells (Figure 1, bottom panel) seeded on fibronectin (FN) and stained for adhesion marker (E-cadherin or β-catenin) and tubulin, we could observe how, as previously reported (Plestant et al., 2014), microtubules cannot reach β-catenin-labeled cell–cell contacts in C2C12 cells (zoom in image in Figure 1, bottom panel), while they could extend to the plasma membrane at points of E-cadherin-mediated adhesions in epithelial cells (Eph4 cells in zoom in image in Figure 1, top panel and Supplementary Figure 2), as previously reported for other cell types (Stehbens et al., 2006; Fuentes and Arregui, 2009). This is probably due to the impedance formed by the actin fibers, as previously demonstrated in C2C12 cells by Plestant et al. (2014), but it does not seem to be the case in MDCK cells (Supplementary Figure 2) where microtubules are in such close contact with the adhesion, that in some points, they almost seem to cross over the junctional actin between neighboring cells (zoom in image in Supplementary Figure 2). The results from microtubule organization around cadherin-mediated adhesion could suggest for the associated extensions of free-ended ER tubules to follow the same tracks, thus, to confirm the extension of ER to the E-cadherin-mediated adhesions, we made use of cells seeded on fibronectin and of a previously characterized system of cells seeded on a planar cadherin (cad-Fc) biomimetic substrate (Supplementary Figure 1). We and others have previously demonstrated that on such substrate, cells form adhesions that nicely resemble actual mature cadherin-mediated adhesions, and they offer an increased optical accessibility also amenable for high-resolution microscopy (Gavard et al., 2004; Bertocchi et al., 2017). Using these two systems, we analyzed ER distribution in epithelial MDCK cells transfected for calnexin-mEmerald, a chaperone protein that resides in the membrane of the ER, and stained for actin or β-catenin, respectively. As shown in Figure 2 (bottom panel), on Ecad-Fc substrate, MDCK cells extend a large circular lamellipodium that contains radial β-catenin-positive adhesion plaques (cadherin-mediated adhesions), mimicking the actual mature cadherin-mediated adhesions of cells seeded on fibronectin (Figure 2, top panel). The ER colocalizes with AJ markers (actin and β-catenin) at sites of adhesion formed between neighboring cells on fibronectin (Figure 2A, top panel) and between cellular E-cadherin and the Ecad-Fc on the biomimetic substrate (Figure 2B, bottom panel). These results confirmed that the ER does indeed extend to AJ sites in epithelial cells.

### Capability of the ER to Reach Cadherin-Mediated Adhesions Depends on Their Tensional State

**FIGURE 3:**
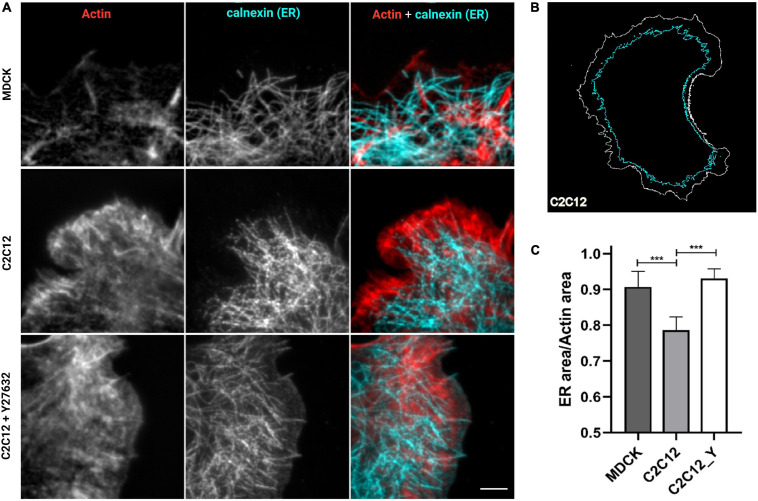
ER is directed toward cell–cell contacts in MDCK cells but are maintained at a distance in C2C12 cells. Release of tension by Y-27632 allows the ER to reach the adhesion similar to MDCK cells. **(A)** Cells transfected for calnexin-mEmerald (for ER identification, in cyan in the images) were plated on cad-Fc biomimetic substrates, left untreated (MDCK and C2C12) or treated with Y compound (Y27632, 10 μM for 1 h), and marked for actin (red) and ER (cyan). Only a small amount of ER (cyan) penetrated the cadherin-mediated adhesion areas of C2C12 cells spread on Ncad-Fc, whereas ER occupies the whole periphery of MDCK cells spread on Ncad-Fc. Images were acquired with a 60x objective, 1.49NA Plan-Apo (Nikon). Scale bar, 5 μm. **(B)** Exemplary contour of the total cell area versus the area occupied by ER. **(C)** The fraction of the whole cell surface occupied by the ER network in MDCK cells, C2C12 cells, and C2C12 treated with Y-27632. The data show the mean ± SD. (*n* = 8 cells analyzed per condition). ***P < 0.0001 (unpaired Student’s *t*-test).

In our previous work (Bertocchi et al., 2017), we have shown that vinculin is a molecular clutch that regulates mechanotransduction at AJs; it is kept in a compact conformation in E-cadherin-mediated adhesion (in MDCK epithelial cells) by dephosphorylation mediated by PTP1B. In N-cadherin-mediated adhesions (in C2C12 cells), vinculin instead is in an extended conformation, possibly due to a higher degree of tension of these cell type and to a lower amount of PTP1B localized at the adhesions. To verify whether the presence of PTP1B at adhesions might be due to the ER’s capability of extending into the adhesion in a cadherin-type specific fashion, we assessed ER structural differences in the two different cell types (MDCK and C2C12 cells) and corresponding cadherins, by using cells transfected with calnexin-mEmerald seeded on cadherin biomimetic substrate for E- and N-cadherin (Ecad-Fc and Ncad-Fc, schematic in Supplementary Figure 1), respectively, and stained with phalloidin (for actin) (Figure 3). We observed that, while in MDCK cells the ER can reach all the way to E-cadherin-mediated adhesions, in C2C12 cells it is not able to penetrate N-cadherin-mediated adhesions (very few extensions appear to protrude till the adhesions), probably due to the same actin arc that impedes the microtubule extension (Plestant et al., 2014). On the same line, we have previously demonstrated that modulation of tension by pharmacological treatment could induce vinculin conformational change, in a similar way as with direct modulation of phosphorylation. To test whether release of tension of the actomyosin cytoskeleton could allow for the ER to extend into the N-cadherin-mediated adhesions, we treated C2C12 with the ROCK inhibitor Y-27632 (10 μM, 1 h). To quantify this effect, we used an extension index determined by the fraction of cell surface occupied by the ER network over the total cell area (as represented in Figure 3B), as analyzed by an IDL-based software. As seen in Figure 3C, following the treatment with Y-27632, these cells show an increased ER area (from 0.79 ± 0.04 in C2C12 not treated, *n* = 6, to 0.93 ± 0.03 in C2C12 after Y27632, *n* = 8), similar to MDCK cells (0.91 ± 0.04, *n* = 6), implying that when tension is released, the ER can reach the adhesion site also in C2C12 cells (Figure 3A lower row and 3C). This suggests a potential relation between ER conformation and PTP1B localization at the adhesion, as through its connection to the ER, the phosphatase could reach cadherin-mediated adhesions in the absence of tension (in MDCK cells). This is not the case in C2C12 cells, as PTP1B is impeded from reaching mature N-cadherin-based adhesions by a structural conformation of the ER. This difference in ER architecture around the different cadherins (E- and N-) in the two cell types is strictly related to the tensional state of the two different types of cadherin-mediated adhesions.

### PTP1B Localizes at Cadherin-Mediated Adhesions

**FIGURE 4:**
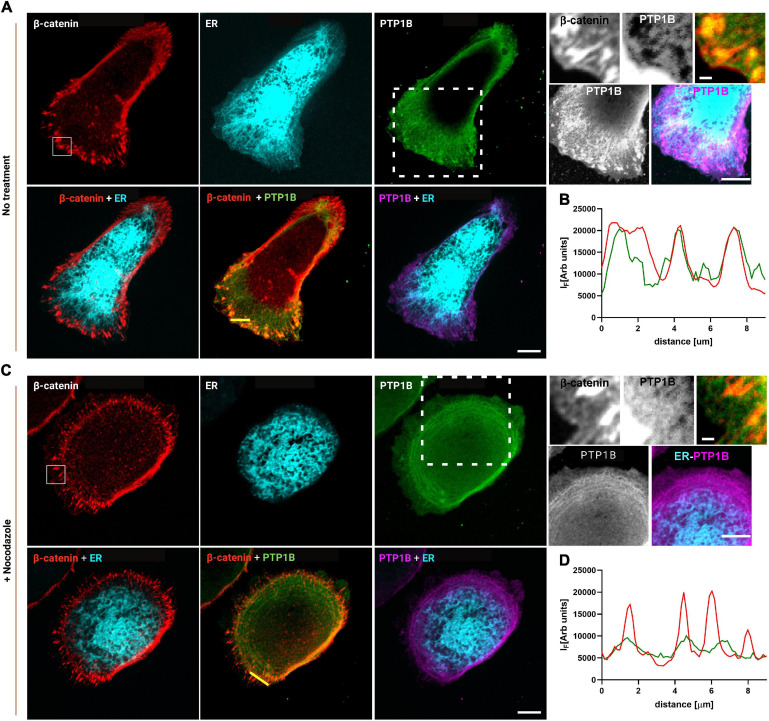
PTP1B colocalizes with β-catenin at E-cadherin-mediated adhesions and is associated with the ER. MDCK cells transfected with calnexin-mEmerald seeded on E-cadherin biomimetic substrate in control **(A)** and after 1 h of nocodazole (10 μm) treatment **(C)**. Top panel of **(A,C)**, representative images of MDCK cells transfected with calnexin-mEmerald (for ER identification, in cyan) and stained for β-catenin (red), and PTP1B (green). In the zoom image (white box in the β-catenin image), the colocalization of β-catenin (red) and PTP1B (green) is visible. In the enhanced contrast image (white dotted box in the PTP1B image), the colocalization of PTP1B (purple) and ER (cyan) is visible. Bottom panel of **(A,C)**, merged images for β-catenin/ER, β-catenin/PTP1B, and ER/PTP1B. In **(B,D)**, line profiles of the dotted lines in PTP1B/β-catenin merged images, for β-catenin and PTP1B, respectively. Images were acquired by spinning disk confocal microscope with a ×60 objective, 1.49NA Plan-Apo (Nikon). Scale bar, 10 μm.

Based on these results, the junctional ER organization in MDCK cells could potentially be the means by which PTP1B can reach cadherin-mediated adhesions. To assess the presence of PTP1B at E-cadherin-mediated adhesions, we seeded MDCK cells on E-cadFc substrate and stained them for PTP1B and β-catenin, as a marker for AJ. As shown in Figure 4, we could indeed confirm by immunofluorescence the localization of PTP1B along the ER (enhanced contrast image in Figure 4, corresponding to the white dotted box in the PTP1B image) and to regions of β-catenin-labeled cell–cell contact (zoom in image in Figure 4, corresponding to the white box in the β-catenin image). As indicated in the exemplary line profiles in Figure 4B, PTP1B clusters appear to colocalize with β-catenin-labeled adhesions, corresponding to the portion of PTP1B ready to engage in enzymatic substrate binding at adhesion. This might be possible if PTP1B is cleaved from the ER, which in other cell types has been reported to be achieved by the action of the calcium-dependent, non-lysosomal cysteine protease, calpain (Frangioni et al., 1993). Disruption of the microtubule network by treatment with nocodazole (10 μM, 1 h) (Figure 4C), unleashes Rho-GEF-H1 microtubule-associated guanine nucleotide exchange factor to activate RhoA with consequent enhancement of contractility and AJ tension (Chang et al., 2008). Such treatment induced a retraction of the ER network toward the cell center, as a consequence of the high interdependence between microtubules and ER (Terasaki et al., 1986; Terasaki and Reese, 1994). Furthermore, the reduction of ER extensions to cadherin-mediated adhesions (marked with β-catenin, in Figure 4) in cells treated with nocodazole, matches the decreased localization of PTP1B at sites of adhesions. These results further suggest that junctional tension controls the ER network extension to cadherin-mediated adhesions and that PTP1B localization requires the association with the ER to reach the adhesion sites.

### Integration of Biochemical Signals by Vinculin Regulates Mechanical Properties of Adherens Junctions

**FIGURE 5:**
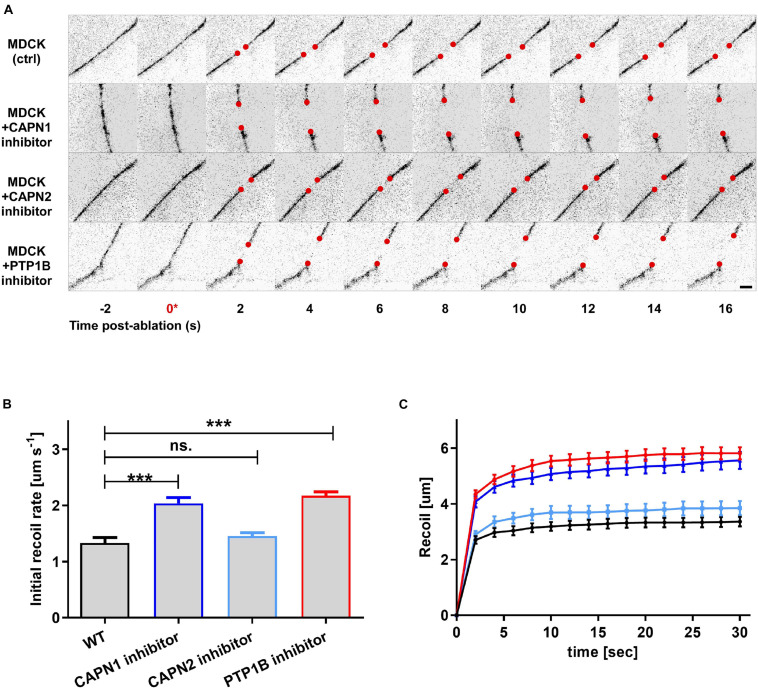
Integration of biochemical signals by the vinculin molecular clutch is regulated by PTP1B dephosphorylation, modulated by calpain cleavage. MDCK cells were cultured on fibronectin-coated substrate to form confluent monolayers, with cell–cell contacts labeled by ZO1-mEmerald. Cells were treated with pharmacological inhibitors as indicated. **(A)** Montage of consecutive frames (interval: 2 s) shown with junction excision at *t* = 0 s (asterisk). Recoiling edges of the junctions (red circles) were used to quantify the recoil trajectory in **(B,C)**. Untreated MDCK (ctrl) were compared with MDCK cells treated with calpain 1 (CAPN1) inhibitor (ALLN, 50 μM for 1 h), calpain 2 (CAPN2) inhibitor (ALLM, 50 μM for 1 h), and PTP1B inhibitor (RK682, 10 μg/ml for 1 h). Scale bar, 5 μm. Junction recoil **(B)** and initial recoil rate **(C)** upon laser ablation of native cell–cell junctions in MDCK epithelial monolayer (wt) and with the indicated pharmacological treatments. Colors of plots in **(C)** correspond to bar graphs in **(B)**. Ablation occurred at 0 s. Images were acquired by Nikon A1R MP laser scanning confocal microscope with 60x obj 1.49 NA. Error bars: S.E.M. *n* values: 15 (ctrl); 17 (+ RK682); 17 (+ ALLN); 11 (+ ALLM). ***P < 0.0005.

To infer about the relationship between junctional tension and vinculin conformational change mediated by PTP1B, we used laser nanoscissors (Hara et al., 2016) to probe tension at cell–cell contacts (Bertocchi et al., 2017). We measured the dynamics of recoil following junction scission in MDCK monolayers treated with inhibitors for PTP1B (RK682) or calpain [a protease previously reported to cleave PTP1B from ER (Frangioni et al., 1993)] (ALLN for calpain-1 and ALLM for calpain-2) as compared with the control (Figure 5). As previously reported, inhibition of vinculin dephosphorylation by treatment with RK682 (PTP1B inhibitor 10 μg/ml, 1 h) can lead to an increase of tension at AJs, probably due to the dislocation of PTP1B and engagement of mechanosensitive Abl kinase (Raghavan and Vasioukhin, 2020; Yu and Zallen, 2020). In agreement with this, our results (Figures 5B,C) show higher initial recoil rates after treatment with calpain-1 inhibitor (slope 0.09 ± 0.009; *P* < 0.0001, *n* = 17) relative to non-treated control (slope 0.05 ± 0.006; *n* = 17), similar to PTP1B inhibition (slope 0.09 ± 0,009; *P* < 0.0001, *n* = 17), indicative of similar levels of tension; inhibition of calpain-2 instead resulted in lower recoil rates (slope 0.06 ± 0.008; *p* = NS, *n* = 11), similar to non-treated control. Our results suggest that calpain-1, and not calpain-2, is involved in maintenance of low junctional tension in E-cadherin-mediated adhesions, similarly to PTP1B, suggesting a role for calpain-1 in PTP1B localization at adhesion, by mediating its cleavage from the ER (as previously reported by Frangioni et al., 1993) or by an unknown indirect effect on other proteins at AJ.

### Effect of Calpain Inhibition on Z-Localization of Vinculin C-Terminus

**FIGURE 6:**
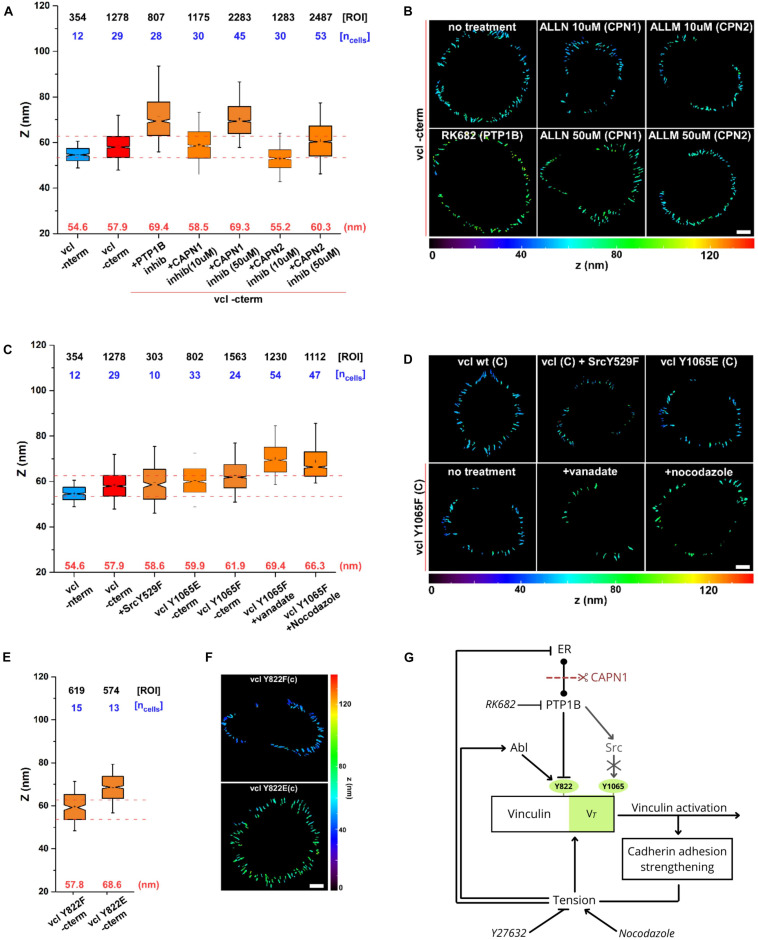
Calpain, through activation of PTP1B, regulates vinculin conformational change. Scanning angle interference microscopy for vinculin Z-position in MDCK cells treated as follows: **(A,B)** no treatment, with PTP1B inhibitor (RK682 10 μg/ml), with calpain-1 (CAPN1) inhibitor (ALLN, 10 and 50 μM), and with calpain-2 (CAPN2) inhibitor (ALLM, 10 and 50 μM); **(C,D)** in MDCK cells transfected with dominant active SrcY529F mutant and in MDCK vinculin KD cells with vinculin phosphomimetic mutant Y1065E and non-phosphorylatable mutant Y1065F, not treated and after drugs treatment (vanadate 10 μM and nocodazole 10 μM, 1 h). **(E,F)** Z-position of vinculin phosphomimetic mutant Y822E and non-phosphorylatable mutant Y822F. **(A,C,E)** Notched box plots for the Z-position of vinculin in E-cadherin-based adhesions. Notched box plots indicate first and third quartiles, median, and confidence intervals; whiskers, 5th and 95th percentiles. Median center values are indicated below each box plot (red). *n* values are shown above each box plot and indicate the numbers of adhesions (number of ROIs, black). Numbers of cells are indicated in blue. Red dotted lines indicate 5th and 95th percentiles of vinculin wt C-terminus for reference. **(B,D,F)** Topographic maps of protein Z-positions (nanometers) of the indicated proteins in E-cadherin-based adhesions of MDCK cells. Images were acquired by TIRF microscope with a 60x objective, 1.49NA Plan-Apo (Nikon). Scale bar, 5 μm. **(G)** Diagram showing the dual mechanism by which tension can modulate vinculin conformational switch.

At last, to evaluate the direct effect of calpain on vinculin conformation, we used scanning angle interference microscopy (SAIM) to measure the Z-localization of vinculin C-terminus (Figure 6) after treatment with PTP1B and calpain inhibitors. As seen in the plot in Figure 6A, and in the corresponding color-coded Z-map in Figure 6B, vinculin C-terminal Z-position after calpain-1 inhibition (ALLN (50 μM, 1 h)) shows a significant upshift to *Z*_center_ = 69.3 nm with respect to the control (*Z*_center_ = 57.9 nm) suggestive of an open vinculin configuration, similarly to what we observe when adding PTP1B inhibitor (*Z*_center_ = 69.4 nm). Lower concentrations of calpain-1 inhibitor (ALLN 10 μM, 1 h), on the other hand, seem to have no effect on the position of the vinculin C-terminus and consecutively on vinculin conformation, as we observe a Z-height (*Z*_center_ = 58.5 nm) similar to control and to calpain-2 inhibitor (ALLM) at any concentration tested, 10 μM (*Z*_center_ = 55.2 nm) and 50 μM (*Z*_center_ = 60.3 nm). These results establish a potential relationship between calpain-1 and maintenance of low junctional tension in E-cadherin-mediated adhesions, where calpain-1 could modulate vinculin conformation (and activation) through direct PTP1B cleavage from the ER, or through a still unexplored pathway.

### Effect of PTP1B on Vinculin Conformation Is Src Independent

As previously reported, PTP1B has been shown to be upstream of Src kinase in several cell models, and is capable of dephosphorylating the inhibitory Y529 site in Src for its activation (Yip et al., 2010). Moreover, Src kinase has been reported to phosphorylate vinculin on tyrosine residues 100 and 1065, promoting vinculin conformational activation in platelet focal adhesions and regulating cellular force transmission (Cheng et al., 2001; Arias-Salgado et al., 2005). To infer about a possible role of Src downstream of PTP1B in AJs, we examined the direct effect of Src kinase on vinculin conformation. For this purpose, MDCK cells were transfected with a dominant active SrcY529F mutant, and MDCK vinculin KD cells with vinculin phosphomimetic mutant Y1065E and non-phosphorylatable mutant Y1065F. As seen in the notched box plot in Figure 6C (and in the respective Z-maps in Figure 6D), neither the active mutant for Src nor the phosphomimetic or non-phosphorylatable vinculin mutants induce a difference in vinculin conformation (*Z*_center_ = 58.6, 59.9, and 61.9 nm, respectively), suggesting that Src is not involved in the modulation of vinculin conformational change (as previously reported in Bertocchi et al., 2017). To exclude a potential involvement of Src in vinculin conformational change, we used drugs to induce tension and/or chemical stimulation [vanadate (100 μM, 1 h) and nocodazole (10 μM, 1 h)] in combination with the non-phosphorylatable mutant Y1065F. As expected, treatment of non-phosphorylatable vinculin mutant Y1065F with nocodazole or vanadate both promoted a significant upshift of vinculin C-terminal Z-position (*Z*_center_ = 66.7 and 69.4 nm, respectively), excluding any potential participation of Src in vinculin conformational changes. As a control, we also tested vinculin phosphomimetic mutant Y822E and non-phosphorylatable mutant Y822F in MDCK vinculin KD (Figures 6E,F), known to be the residue phosphorylated by Abl kinase at AJs (Bays et al., 2014). As indicated by the box plot in Figure 6E and the corresponding Z-map in Figure 6F, Y822E phosphomimetic vinculin mutant shows an extension of the C-terminal Z-position (*Z*_center_ = 68.6 nm) similar to what was observed with PTP1B inhibition (*Z*_center_ = 69.4 nm; Figures 6A,B), suggesting its implication in vinculin conformational change.

## Discussion

Our study sheds new light on the participation of junctional ER structural organization in mechanotransduction at cadherin-based adhesions by modulating vinculin conformation (Figure 6G). We suggest that the structure of the junctional ER extensions, and linked PTP1B, to AJs is tension dependent. This introduces an additional level of complexity to the structural framework of AJ dynamics, and in the modulation of mechanotransduction of AJs mediated by vinculin conformational state. It may as well explain how the difference observed in phosphorylation and conformational switch of vinculin in diverse cell types is intimately intertwined with structural differences in the ER/microtubule organization around E- and N-cadherin-mediated adhesions.

In our previous paper (Bertocchi et al., 2017), we mapped the multicompartment nanoscale architecture of cadherin-mediated adhesions, elucidating a plasma membrane-proximal cadherin-catenin layer segregated from the actin cytoskeletal compartment, bridged by an interface zone containing vinculin. Vinculin, anchored with its N-terminus to open α-catenin (Yonemura et al., 2010), upon activation would extend ∼30 nm to bridge the cadherin-catenin and actin compartments, while modulating the nanoscale positions of actin regulators. Differently from what has been observed in focal adhesions, where vinculin conformation is regulated by Src on Y1065, Y100, S1033, and S1045, and is seemingly necessary for adhesion growth and maturation (Golji et al., 2012; Huang et al., 2014), in AJ, vinculin conformational switch requires tension and tyrosine phosphorylation regulated by Abl kinase and PTP1B phosphatase (Bays et al., 2014; Bertocchi et al., 2017). Furthermore, we have observed that in lower tensional E-cadherin-based adhesions (Yonemura et al., 2010), vinculin adopts a compacted partially unfurled conformation, maintained by PTP1B dephosphorylation, whereas, in the higher tensile N-cadherin-based adhesions [expressed in tissues like muscle (Wang et al., 2015), vascular (Sun et al., 2014, 2017), cardiac (Piven et al., 2011), or retinal tissue (Lilien et al., 2002; Masai et al., 2003)], vinculin may be fully extended due to both higher contractility and probably lower PTP1B localization at the site of adhesion. Still, it remained unknown whether this difference could reflect E-/N-cadherin specificity and if it could be due to a physical inaccessibility of PTP1B to the N-cadherin-based adhesions, possibly related to a structural difference of the ER/microtubules network in the different cell types and their tensional states.

Previous reports indicated that the ER is in close proximity to the plasma membrane at regions of cell–cell contact, and that ER-anchored PTP1B engages with substrate(s) at these locations in specific cell types (Hernández et al., 2006). In the present study, we firstly demonstrated that the ER extensions could reach the site of E-cadherin-adhesions in epithelial cells (Figure 2), probably guided by their interdependence with microtubules (Figure 1, Top panel), similarly to what was reported previously (Fuentes and Arregui, 2009). Subsequently, by comparing epithelial cells (EpH4 and MDCK cells) with myoblasts (C2C12 cells), that present a different type of cadherin (N-cadherin), we could observe a striking difference in the organization of the microtubules and the ER around the two different types of cadherin (E- and N-cadherin), in the two different cell types (Figures 1–3). Indeed, microtubules and ER could extend to the E-cadherin-mediated adhesions in epithelial cells (in both epithelial monolayers (Figure 2, top panel) and in cells spread on the biomimetic E-cad-Fc substrate (Figure 2, bottom panel, Figure 3), while in C2C12 cells, both the microtubules (Figure 1, bottom panel) and the ER (Figure 3) showed a much lower capability of penetration to the adhesions. These results are well in accordance with the findings by Plestant et al. (2014), who elegantly showed that in C2C12 cells spread on N-cad-Fc substrate, the microtubules cannot penetrate the tangential actomyosin arc present at the rear of the lamellipodium, revealing an obstructive effect of the tangential actin bundles on microtubule penetration into the adhesion area. This barrier effect exerted by the actin arc on microtubule penetration appears to be absent in epithelial cells (Supplementary Figure 2), and it could represent a possible explanation for the difference observed in the microtubules and ER organization in the two different cell types. Interestingly, our release of tension by ROCK inhibitor, which would induce a relief from this actin impediment, allows for the ER to fully extend into N-cadherin-mediated adhesions (Figure 3). This difference in the structural organization of the ER network around cadherin-mediated adhesions in the two cell types (epithelial vs. myoblasts) could be a possible explanation for the observed difference in vinculin conformation (compact vs. extended, respectively) in the two cell types and the associated specific-cadherin type (E-cadherin and N-cadherin, respectively), and their different tensional states (cell related, or mechanically induced by actomyosin contractility). Since vinculin was reported to be maintained in a compact conformation in MDCK cells by PTP1B-mediated dephosphorylation (Bertocchi et al., 2017), we have hypothesized that the ability of the ER to penetrate E-cadherin-mediated adhesions in MDCK could represent the path for PTP1B to reach its substrate (vinculin) in the interface zone. This was indeed confirmed in Figure 4, where, in MDCK cells, PTP1B is shown to run along the ER (enhanced contrast image in Figure 4A) and to cluster and colocalize with β-catenin adhesions (Figures 4A,B), while it is lost from the adhesion sites after increase of tension by nocodazole (Figures 4C,D). This is clearly related to nocodazole-induced microtubule depolymerization and the subsequent effect on the ER network, which shrinks and bends on itself far away from the lamellipodium, similarly to what was previously observed in C2C12 cells (Plestant et al., 2014), eventually blocking the localization of PTP1B to the adhesion. The relation between tension and PTP1B dislocation was anticipated in our previous publication (Bertocchi et al., 2017), where we demonstrated that short inhibition (1 h) of PTP1B by RK682 could induce an increase in junctional tension in epithelial cells, similar to treatment with nocodazole [known for its effect on cell contraction and tension at AJs (Chang et al., 2008; Ito et al., 2017)]. This was further corroborated by knockdown of PTP1B by siRNA in EpH4 cells (Supplementary Figure 3), where we could indeed observe an increase in the E-cadherin fluorescence intensity (indicative of increased tension) at adhesions (which also present some degree of morphological change), similarly to what is observed after nocodazole treatment (Supplementary Figures 3B,C).

Although PTP1B has enzymatic activity in its ER-bound state (Haj et al., 2002; Boute et al., 2003; Romsicki et al., 2004; Yudushkin et al., 2007), it can increase up to twofold when cleaved from the ER (Frangioni et al., 1993; Hernández et al., 2006). PTP1B cleavage from the ER has been previously demonstrated to be catalyzed by the calcium-dependent neutral protease calpain (Frangioni et al., 1993); furthermore, calpain-1 inhibitor, ALLN, has been demonstrated to inhibit the calpain/PTP1B axis in endothelial cells (Zhang et al., 2017). In accordance with this, our results indicate a correlation between PTP1B and calpain-1. Inhibition of calpain-1, similarly to PTP1B inhibition induces increase in junctional tension (Figure 5) and provokes a switch of vinculin to an extended conformation (Figure 6A). This implies that the inhibition of calpain-1 can affect junctional tension by modulation of vinculin conformation through cleavage of PTP1B from the ER, or through a still unexplored pathway possibly involving other adhesion proteins. On the other hand, inhibition of calpain-2, which has been reported to be involved in invadopodia regulation through a PTP1B/Src signaling pathway (Cortesio et al., 2008), revealed no significant effects on tension nor on vinculin conformation. Thus, we can disregard a role for a calpain-2/PTP1B/Src pathway in the regulation of mechanotransduction of AJ through its effect on vinculin. This was further confirmed by our results with SAIM using constitutively active Src or phosphomimetic and non-phosphorylatable vinculin mutants for Src phosphorylation (Figure 6B).

In conclusion, we propose a mechanism (Figure 6C) by which tension can modulate vinculin conformational switch, either by direct mechanical stretch induced by actomyosin contractility or by modulating the structural arrangement of the junctional ER network that controls PTP1B phosphatase’s localization at AJ and, eventually, vinculin activation state.

## Data Availability Statement

The original contributions presented in the study are included in the article/Supplementary Material, further inquiries can be directed to the corresponding author/s.

## Author Contributions

CB and MJ-I: data production and analysis. AR and MC: data analysis supervision. CB, YT, and PK: experimental design. MJ-I and MR: literature screening. CB and PK: study conception and project oversight. PK, YT, and CB: provided material and infrastructure. All authors edited and revised the manuscript drafts and approved the final version of the manuscript.

## Conflict of Interest

The authors declare that the research was conducted in the absence of any commercial or financial relationships that could be construed as a potential conflict of interest.

## Abbreviations

AJ: adherens junctions
ER: endoplasmic reticulum
PTP1B: protein-tyrosine phosphatase 1B SAIM scanning angle interference microscopy.
