# Supplementary material for: Junctional ER Organization Affects Mechanotransduction at Cadherin-Mediated Adhesions
Source: Front Cell Dev Biol. 2021 Jun 17;9:669086. doi: 10.3389/fcell.2021.669086 (PMC8247578; doi:10.3389/fcell.2021.669086)
Supplement: Supplementary file 1 [file Data_Sheet_1.docx]

**Junctional ER organization affects mechanotransduction at cadherin-mediated adhesions.**

**M. Joy-Immediato^1^, M. J. Ramirez^1^, M. Cerda^2,3^, Y. Toyama^4,5^, A. Ravasio^6^, P. Kanchanawong^4,7*^, C. Bertocchi^1*^.**

^1^ Laboratory for Molecular Mechanics of Cell Adhesion, Department of Physiology, Faculty of Biological Sciences, Pontificia Universidad Católica De Chile, Santiago, Chile.

^2^ Institute of Biomedical Sciences, Faculty of Medicine, Universidad de Chile, Santiago, Chile.

^3^ Center for Medical Informatics and Telemedicine, Faculty of Medicine, Universidad de Chile, Santiago, Chile.

^4^ Mechanobiology Institute, National University of Singapore, Singapore.

^5^ Department of Biological Sciences, Faculty of Science, National University of Singapore, Singapore.

^6^ Institute for Biological and Medical Engineering, Schools of Engineering, Medicine and Biological Sciences, Pontificia Universidad Católica de Chile, Santiago, Chile.

^7^ Department of Biomedical Engineering, Faculty of Engineering, National University of Singapore, Singapore.

**^*^ Correspondence:**

Cristina Bertocchi [cbertocchi@bio.puc.cl](mailto:cbertocchi@bio.puc.cl)

Pakorn Kanchanawong [biekp@nus.edu.sg](mailto:biekp@nus.edu.sg)

**Supplementary Figure 1: Model of planarized biomimetic (N- or E-) cadherin-Fc substrate.** Silanized glass coverslips or silicon wafers were coated by anti-F_c_ IgG, followed by purified cadherin-F_c_ chimeric protein. Cells were seeded onto the substrate in absence of serum to avoid extracellular matrix deposition so that adhesions were formed primarily via cellular cadherin engagement to the substrate-bound cadherin-F_c_. See material and methods section for details.


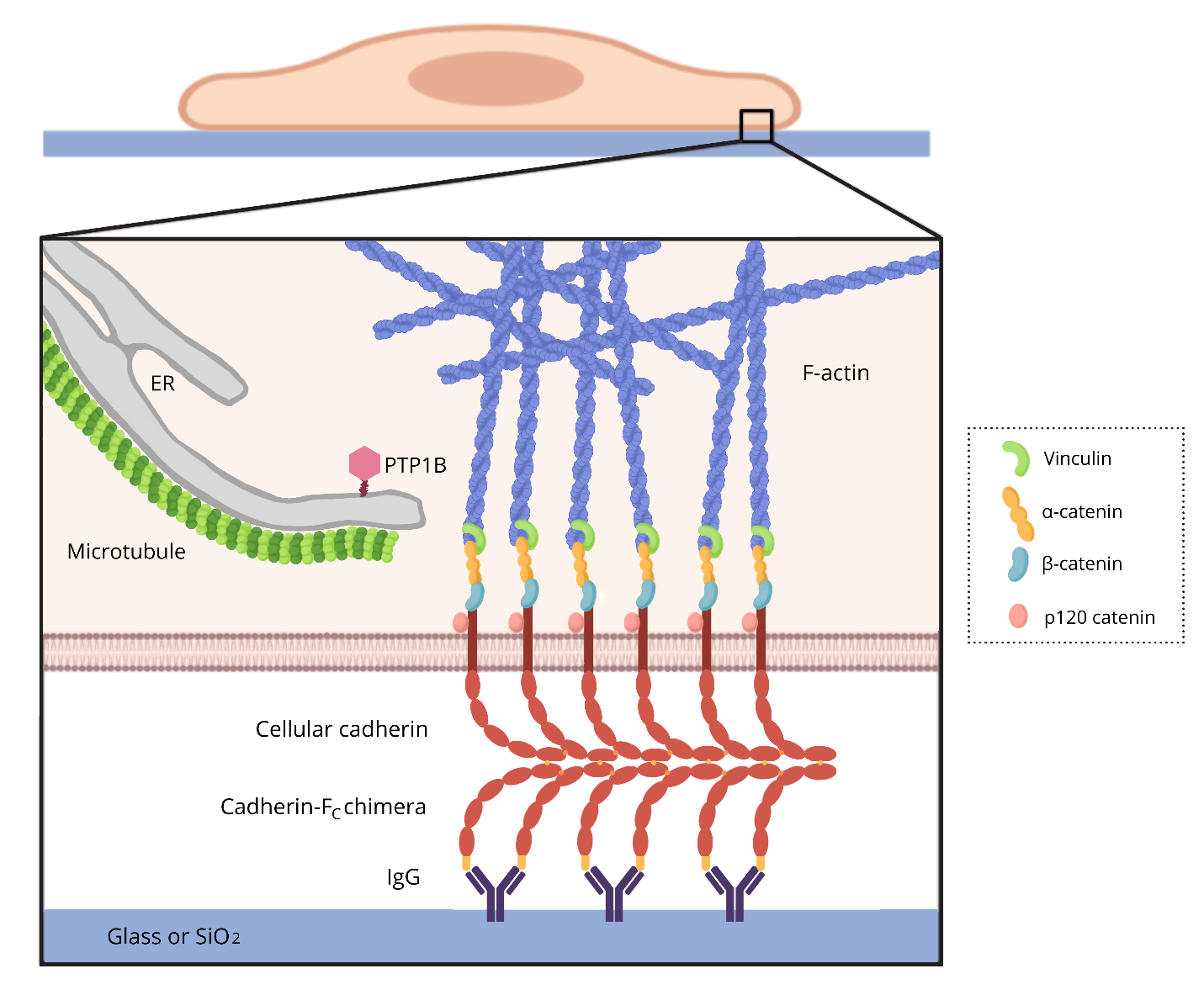


**Supplementary Figure 2: Microtubules and actin in epithelial cells.** Eph4 cell monolayers labelled for tubulin (cyan) and Actin (red). Images represent microtubule staining alone, actin and the merge image. In the zoom image (white box in the Actin image on top panel) it is visible the overlapping of the microtubule network (cyan) with Actin at adhesion (red). The microtubules (cyan) are in such close contact with the adhesion, that at some points, they almost seem to cross over the junctional actin (in red) between neighboring cells. Images were acquired by Spinning disk confocal microscope with a 100× objective, 1.49NA Plan-Apo (Nikon). Scale bars 10µm.

**
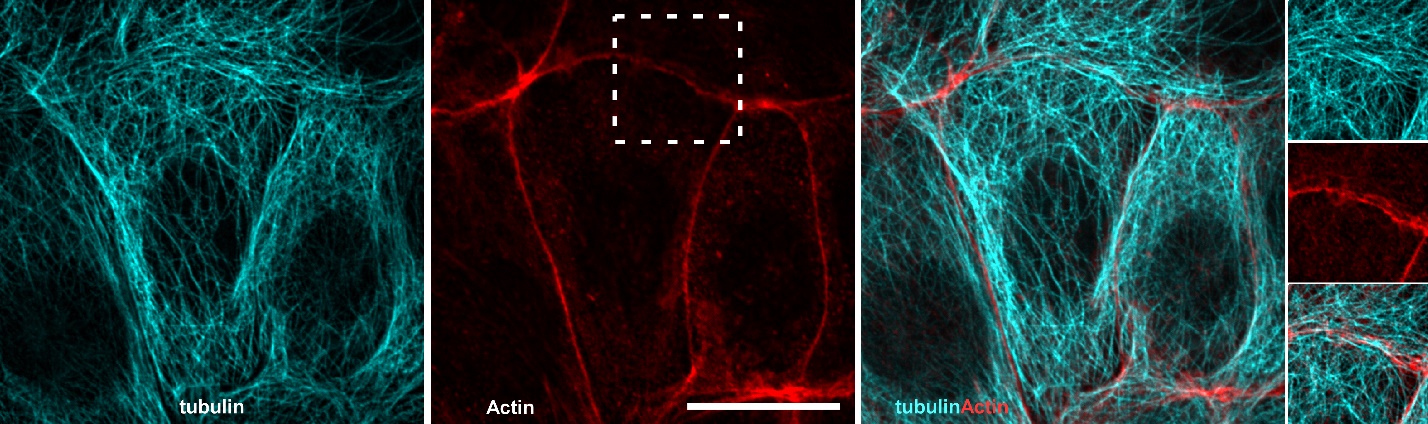
**

**Supplementary Figure 3: Effect of PTP1B knockdown on E-cadherin mediated adhesion.** To evaluate the effect of PTP1B silencing on E-cadherin-mediated adhesions, we have compared the intensity of cadherin (used as marker for adherens junctions) fluorescence in NegsiRNA control cells (transfected with scrambled siRNA), the same cells treated with nocodazole (known to induce tension at the AJ), and siRNA PTP1B cells. EpH4 cells were transfected in suspension with a mouse scrambled siRNA (NegsiRNA control) or siRNA targeting PTP1B using SMARTpool concentration of 50 nM. Control cells were either left untreated or treated with Nocodazole (10 μM, 1h). After fixation (24h after transfection), cells were imaged by Spinning disk confocal microscope with a 60× objective, 1.49NA Plan-Apo (Nikon). **(A)** Images presented are z-average projections, background subtracted. Scale bar 10µm. **(B)** Bar chart of the fluorescence intensity of cadherin staining of the NegsiRNA control, NegsiRNA treated with nocodazole (10 μM 1 hour), and PTP1B siRNA. All images have been segmented by Trainable Weka Segmentation Plugin in Image J, background corrected, and quantified for the intensity of E cadherin at the adhesion. Results are normalized for NegsiRNA (n=3 triplicates for each condition); *: p < 0.05; **: p < 0.005 **(C)** Exemplary line profiles for each of the conditions in (**A**). The line in (**A**) indicates the line used for the measurement of the line profiles in (**C**).

**
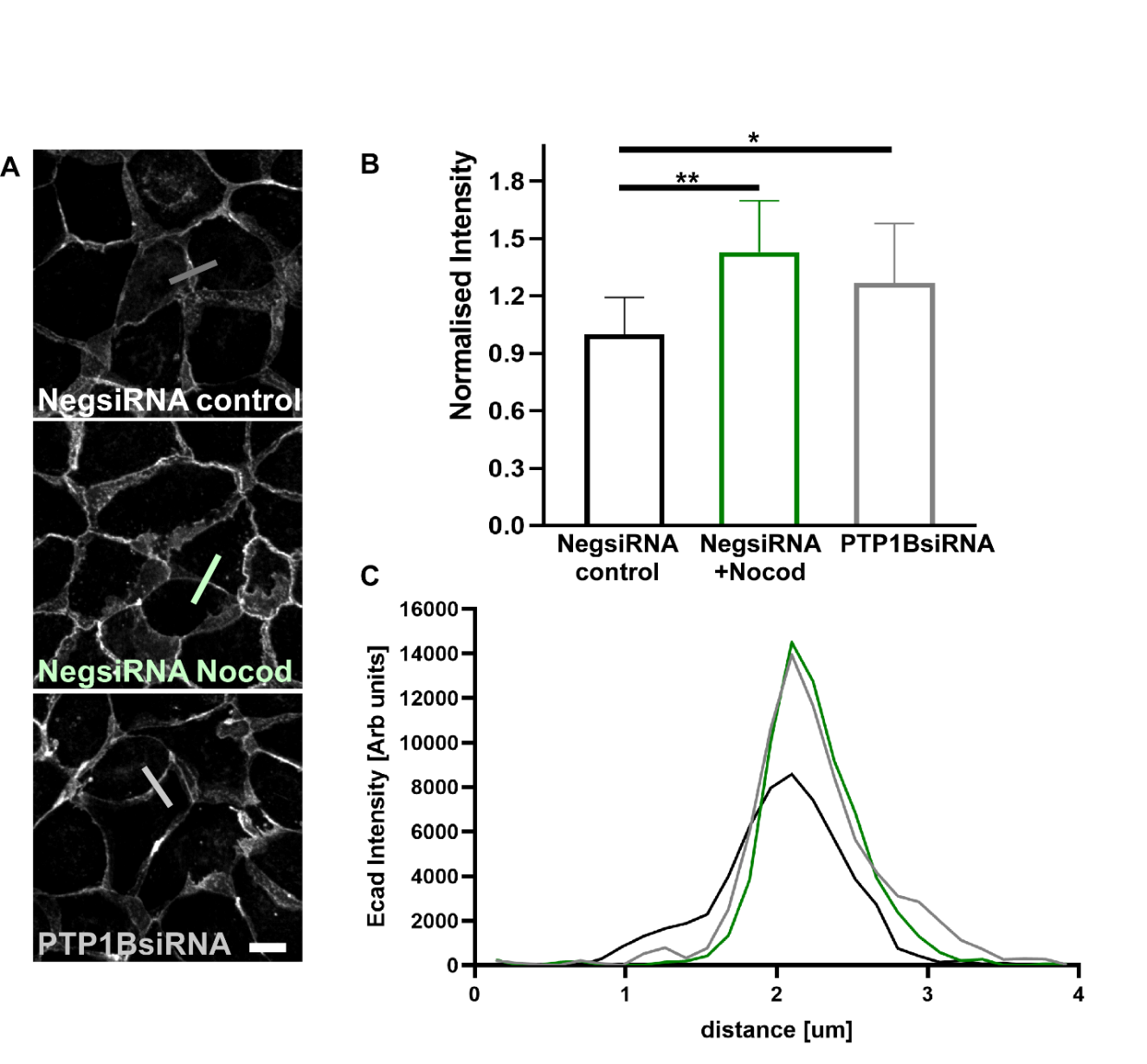
**

**Supplementary Table 1: RNA seq data for gene expression of phosphatases reported to be blocked by RK682, in MDCK and EpH4.**
